# Low noise, open-source QEPAS system with instrumentation amplifier

**DOI:** 10.1038/s41598-019-38509-7

**Published:** 2019-02-12

**Authors:** Mateusz Winkowski, Tadeusz Stacewicz

**Affiliations:** Institute of Experimental Physics, Faculty of Physics, University of Warsaw, Pasteura 5, 02-093 Warsaw, Poland

**Keywords:** Optical sensors, Optical sensors, Optical sensors, Infrared spectroscopy, Infrared spectroscopy

## Abstract

Quartz enhanced photoacoustic spectroscopy (QEPAS) is a rapidly developing, ultrasensitive method for trace gas sensing. Adequate electronic amplifier, well matched to the quartz characteristics is crucial for overall system performance. Here we present an open source circuit for QEPAS signal amplification. It consists of a buffer, instrumentation amplifier and digitally controlled gain stage. An experiment showed, that it offers signal to noise ratio of about 23 dB better than commonly used transimpedance amplifier. The use of this circuit provides opportunity to improve QEPAS sensitivity by about one order of magnitude.

## Introduction

Trace gas sensing is an important issue widely used in many applications, like breath analysis, environmental monitoring^1^, industrial processes control^2^, medical diagnosis^3^ and detecting of toxic gases^4^ or the explosives^5^. Laser absorption spectroscopy is useful for the target achieving. Photoacoustic spectroscopy is characterized by ultrahigh sensitivity compared with other methods^6^. In this method the examined gas is treated by laser light, with the wavelength precisely adjusted to the absorption spectrum of the compound of interest. The light, modulated with the frequency *f*, induces periodic changes of temperature and pressure. That causes the sound wave of the frequency *f* in the medium. The wave, detected with a microphone, provides a photoacoustic signal. Its amplitude is proportional to the absorber concentration and the laser beam power. The measurement can be performed with a dynamic range of the absorption coefficient of several orders of magnitude^7^. This approach does not need any optical sensor, therefore it can be used for the wavelength range, where there is a lack of sensitive photodetectors.

## Quartz Enhanced Photoacoustic Spectroscopy

**Figure 1:**
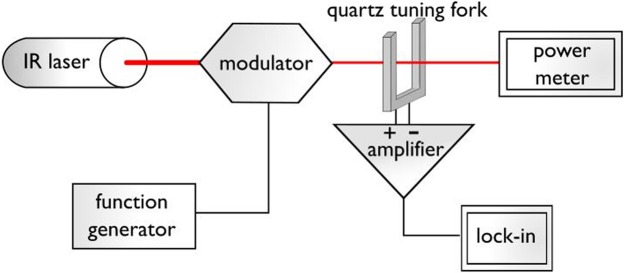
Simplified QEPAS setup.

High sensitivity of photoacoustic approach is crucial for very low gas concentrations detecting. The use of a quartz tuning fork (QTF) as piezoelectric acoustic wave detector is a favourable solution^8^. This method is called quartz enhanced photoacoustic spectroscopy (QEPAS). Scheme of typical QEPAS setup is shown in Fig. 1.

The laser beam is focused between the fork prongs. Asymmetric vibrations of the prongs, induced by the laser light, cause asymmetric electric oscillations which are amplified due to crystal geometry being an acoustic quadrupole^9^. The system quenches symmetric oscillations caused by external sources. Thus, the system provides outstanding noise immunity.

The quartz forks from electronic watches are widely applied here. Their frequency is 32768 Hz, while the Q-factor reaches about 10^4^ in air^10^. Narrow bandpass characteristic of such microphone is another cause of the noise quenching. Small size of the QTF (about 3,2 mm in length, 1 × 0,4 mm in base) provides opportunity to use QEPAS system for investigation of the low gas amount. Low cost of this piezoelectric transducer (less than one dollar) makes them preferable for construction of cheap sensors. QEPAS was already used for detecting of nitric oxide^11^, ammonia^12^ or carbon monoxide^13^ in ppb-concentration.

Although quartz-enhanced photoacoustic spectroscopy is rather novel technique, it is well developed. Many improvements were already presented: with on^14,15^ and off-beam^16^, acoustic microresonators, multiple-quartz solutions^17^, custom crystal detectors^18^, etc. Furthermore, the stimulating signal was examined resulting in 2f^19^ or beat-frequency approaches^20^.

**Figure 2:**
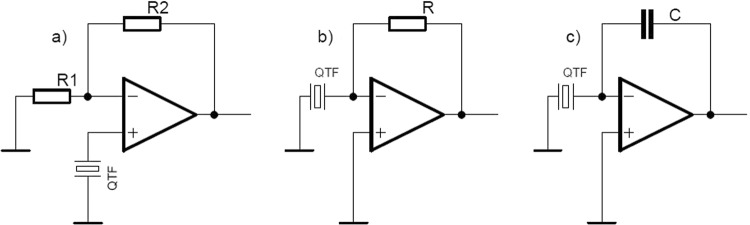
QEPAS amplifier topology (simplified).
- Transimpedance amplifier (Fig. 2b). There is virtually no voltage difference between quartz tuning fork leads in this scheme. Due to that an impact of parallel capacitance of the crystal is minimized. This amplifier is broadly used in almost all QEPAS experiments^21,22^, commonly with the resistance value R = 10 MΩ. As far as it provides fine gain, almost nobody cares about parasitic capacitance, that is always present in the circuit. Assuming QFT capacitance of 1 pF only in parallel with the resistor R it creates a lowpass filter with cut-off frequency about 16 kHz. Therefore the signal is significantly attenuated, since the quartz crystal resonant frequency exceeds 32 kHz.
- Charge amplifier (Fig. 2c). This configuration is preferable when the signal source has capacitive character. Its gain, equal Cs/C, is frequency independent in contrast to transimpedance variant. Quartz tuning fork is working at constant, narrow frequency about 32.768 kHz, therefore there is no problem with frequency caused gain drifting. However a complication appears with obtaining of a gain that is comparable to that one of the transimpedance configuration. It is hard to realize because the feedback capacitor C should have the capacitance of about 0.47 pF, whereas its parasitic capacitance is usually larger.

Quartz tuning fork should operate together with an amplifier that provides proper signal for further measurement (Fig. 1). Despite many papers about QEPAS performance, not much information was given on the amplifier design. It is usually stated that high input impedance amplifier has to be used. Basically three types of amplifier topology can be used:

- Voltage amplifying circuit (Fig. 2a). Its noninverting configuration preserves high input impedance, since the QTF is connected directly to operational amplifier (op-amp).

Electrical properties of the quartz crystal require suitable amplifier topology. That is crucial for obtaining of high signal to noise ratio (SNR) and the high sensitivity. Recent publication^23^ shows, that a usage of voltage amplifying circuit instead of widely utilized transimpedance topology, can significantly increase the system efficiency. Unfortunately, neither the electric scheme nor the printed circuit board were published. In this paper we present a solution based on instrumentation amplifier.

## Overall Amplifier Schematic

**Figure 3:**
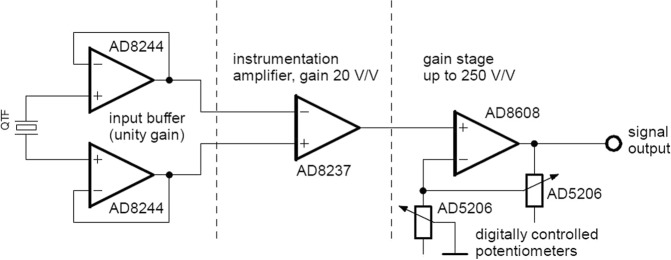
Designed QEPAS amplifier (simplified, one channel).

Simplified scheme of QEPAS amplifier, constructed in our laboratory is shown in Fig. 3. It contains two identical channels, designed for two independent quartz forks. Full schematic and printed circuit board (PCB) fabrication data is available at http://www.fuw.edu.pl/mwinkowski/.

First stage of the system buffers the signal. It should be placed as close as possible in respect to QTF, preferably inside shielded metal case. Due to the impedance transformation the QEPAS system is less vulnerable to the external noise. Buffered quartz fork is treated as low amplitude differential voltage source. In the next stage the instrumentation amplifier provides 20 times gain of differential signal and quenches the common-mode voltage. Last stage allows additional amplification of the low signals. This part provides digitally controlled gain reaching 250 V/V.

## Quartz Tuning Fork – Crystal Characteristics

**Figure 4:**
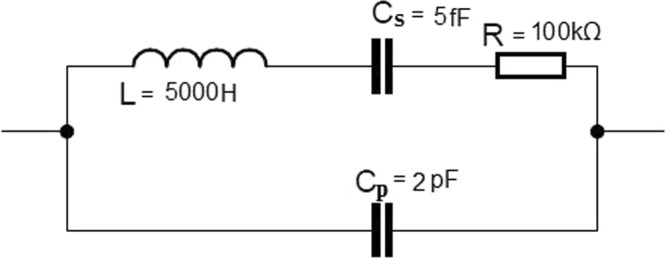
QTF electrical model (approximate values).

As mentioned above, the acoustic wave causes the mechanical vibration of QTF prongs. The physical stress causes electric field, due to the piezoelectricity of the quartz crystal. Therefore quartz tuning fork can be modelled both mechanically (mass, spring, damper) and electrically (resistor, capacitor, inductor). Approximate values of the electric elements are given in Fig. 4 ^24^.

Quartz tuning forks used for the resonators have the charge constant (electric charge produced in the material per unit of the mechanical stress) of 4.6 pC/N^25^, which is low, compared with other piezoelectric crystals. However, its voltage constant (electric charge produced in the material per unit of the mechanical stress) reaches relatively high value of 118 Vm/N.

## Detailed Design

According to mentioned crystal characteristics, treating the QTF as voltage source (Fig. 2a) is more profitable than the using it as current or charge source (Fig. 2b,c). If the crystal signal is particularly weak, one can benefit from instrumentation amplifier application. This kind of voltage amplifying circuit is suitable for the measurement of small differential signal with large common-mode voltage. The ability of easy overall gain change is an important feature for QEPAS system, allowing highest operating range. Our solution provides remote regulation without opening of the metal cover.

### Buffering preamplifier

Quartz tuning fork is especially vulnerable to external noise due to high source impedance. Using of a unity-gain voltage amplifier with high input and low output impedance is the most effective way to reduce it.

Two ways for buffer designing were considered: the selection of dedicated integrated circuit or the use of an operational amplifier in voltage-following configuration. The input signal path should be guarded from the other traces because of high source impedance. Here may be a problem with routing a guard path when using the operational amplifiers in the packages with small footprints (distance between the pins 0.65 mm or less), since the noninverting inputs are typically placed next to the power supply or the ground pin. Thus, the integrated circuit dedicated for the buffer was chosen. Due to application of two separate amplifier channels four inputs are needed. Furthermore, high input resistance, low input bias current, low noise, low voltage offset (and its drift), and matched gain between the channels are crucial for the system effectiveness. AD8244, precision FET input quad buffer was chosen due to its excellent performance characteristics - primarily 10 TΩ input resistance and 0.5 pA input bias current. Besides, its design isolates inputs from low impedance leakage sources^26^.

### Instrumentation amplifier

Instrumentation amplifier solution was chosen due to its ability to extract very low differential signal from high common mode voltage. Such advantage, called common mode rejection ratio (CMRR), reaches over 100 dB for the best integrated circuits. Proper bandwidth, low noise, low voltage offset (and its drift), and gain error were analysed when selecting the integrated circuit. Finally, AD8237 was used in each channel. Its modern design provides 120 dB CMRR and 0.005% gain error^27^.

Special attention has to be paid to input common mode voltage vs. output voltage (also called diamond plot) when selecting the integrated circuit. It is especially important when the system is battery powered with low voltage power supply. In such case AD8237 architecture allows wide input voltage range, which is unique to other instrumentation amplifiers.

### Digitally controlled gain stage

One of the advantages of QEPAS is high linearity in wide measurement range. Our QEPAS amplifier provides variable voltage gain in order to fully benefit from this feature, combining the operational amplifier with AD5206 digital potentiometer. It can be comfortably adjusted with 8-bit precision.

Special care must be taken to the element tolerance when using the digital potentiometer, as it directly affects the gain precision. Nominal absolute tolerance of AD5206 is 30%, according to its datasheet^28^, although matching between the channels is typically 0.25%, i.e. 120 times better. Therefore both (variable) resistors used in the noninverting voltage amplifier are taken from AD5206 integrated circuit for smallest gain error.

### Power supply

The amplifier is powered with single cell to maintain the service convenience. Rechargeable lithium-ion battery was chosen, since it offers high energy density^29^. No switching-mode power regulators are used, in order to avoid introducing their noise into the QEPAS system.

All components were selected to allow working from the battery in its full voltage range: 3.3 to 4.2 V. The amplifier is also compatible with USB standard (5 V). In order to fully exploit the supply voltage, the rail-to-rail operating circuits were applied.

Virtual ground circuit enables operation from single supply, despite the use of instrumentation amplifiers. In another variant (with minor PCB changes) a split power supply (not stabilized or exceeding 5.5 V) is used with two linear voltage regulators: ADP7142 (positive) and ADP7182 (negative).

### PCB design

Proper PCB design is crucial for both performance and functionality. High impedance signal lanes are guarded due to the parasitic capacitances. Power supply path are as thick as possible. Ground plane is also a must-have in precise analogue circuits. Bypass capacitors are placed as close to power pins of the integrated circuits as possible. Small resistors are placed at digital lines to limit the noise affecting the signal paths^30^. Designed circuit provides active shielding, so enclosing it in metal case is advisable.

## Tests and Measurements

A An experiment was run to compare the designed circuit with simple transimpedance topology amplifier. For this purpose, proper QEPAS measuring system used for evaluating the amplifier was built (Fig. 1).

Toptica distributed feedback laser was applied as an infrared light source. Its wavelength was matched to a peak of a strong line in water vapour absorption spectrum (1392.534 nm) using WS6 wavemeter (HighPrecision). Laser beam intensity was modulated with electro-optic modulator (Jena Optics) working with function generator (Tektronix AFG3102) and focused between the QTF prongs. Measured (Ophir Starlite) laser power (without modulation) was 0.5 mW. The generator was precisely tuned to crystal resonant frequency, 32758.3 Hz (nominal QTF frequency changes after opening the vacuum case, due to air viscosity and atmospheric pressure). The signal from the examined circuit was then measured with lock-in amplifier (Stanford Research System SR 830). One second integration time was used. Relative air humidity during the experiment was about 42% and air temperature was 20 °C.

In order to compare amplifiers’ performance, signal to noise ratio (SNR) was measured in two cases: for the designed circuit with overall gain 20 V/V or 1200 V/V, and for ordinary transimpedance amplifier with 10 MΩ feedback resistor, as applied in other publications^7–9^. SNR was calculated as a ratio of voltage signal with laser turned on and off, according to the Eq. (1).

1 $${\rm{SNR}}\,[{\rm{dB}}]={\rm{20}}\,{\mathrm{log}}_{{\rm{10}}}\frac{{V}_{on}}{{V}_{off}}$$

Therefore, calculated SNR is:

- 32.46 dB for ordinary transimpedance amplifier
- 55.56 dB for designed voltage amplifier with gain 20 V/V
- 55.27 dB for designed voltage amplifier with gain 1200 V/V.

The experiment shows that our circuit is characterized by SNR about 23 dB better than the transimpedance amplifier (used in other QEPAS systems). Therefore one can evaluate that our device is able to provide the sensitivity about one order of magnitude better. That was achieved almost without the additional costs. On the other hand, overall QEPAS system expense may be greatly reduced (while keeping its precision), due to lower demand for the laser power. Highest available gain of our electronics does not degrade its parameters, therefore it is suitable for trace gas concentrations.

## Discussion

In order to compare our circuit with already reported QEPAS-based systems, normalized noise equivalent absorption (NNEA) was calculated for both transimpedance and instrumentation amplifier cases. The analysis was done for water vapour detection. Results are collected in Table 1.

**Table 1:** QEPAS-based water vapour detection systems.

QEPAS system	Wavelenght [nm]	NNEA (descending)	QEPAS microresonator
Transimpedance amplifier (tested)	1392.53	8.4 · 10^−8^	No (bare QTF)
Reference^33^	1395.51	1.7 · 10^−8^	Yes, on-beam
Reference^34^	1528.75	8.0 · 10^−9^	Yes, on-beam
Reference^16^	1396.37	5.9 · 10^−9^	Yes, off-beam
Instrumentation amplifier (tested)	1392.53	5.8 · 10^−9^	No (bare QTF)
Reference^35^	1368.60	1.9 · 10^−9^	Yes, on-beam

Such analysis shows that NNEA is typically better for custom QEPAS amplifier than for already reported systems. All systems compared in Table 1, except ours were used with acoustic microresonators (either on-beam or off-beam). Application of the resonator improves SNR (and NNEA) from 7-fold^31^ up to 30-fold^32^. It explains the fact, why NNEA of our transimpedance amplifier based system (Table 1) is about 10 times worse comparing with solutions working with microresonators.

## Summary

Two channel QEPAS amplifier system was constructed. It consists of three parts. Unity gain buffer placed close to the crystal greatly increases the immunity to the external noise. The instrumentation amplifier provides essential gain of the signal. It is further amplified with the third, digitally controlled stage. The circuit is battery powered with single Li-ion cell.

In order to compare the designed system with ordinary transimpedance amplifier QEPAS an experiment was carried out. The circuit is characterized by signal to noise ratio that is about 23 dB better than for commonly used topology, even at the highest gain. High NNEA of 5.8·10^−9^ was achieved despite working with bare QTF (with no microresonator) at atmospheric pressure.

Both electrical schematic and PCB fabrication data is free to download. Full schematic and printed circuit board (PCB) fabrication data is available at http://www.fuw.edu.pl/mwinkowski/.

## Data Availability

Authors declare that all data supporting the findings of this study can be found within the article and its Supplementary Information Files.
