# Author Correction: Low noise, open-source QEPAS system with instrumentation amplifier

**DOI:** 10.1038/s41598-020-57571-0

**Published:** 2020-01-14

**Authors:** Mateusz Winkowski, Tadeusz Stacewicz

**Affiliations:** Institute of Experimental Physics, Faculty of Physics, University of Warsaw, Pasteura 5, 02-093 Warsaw, Poland

Correction to: *Scientific Reports* 10.1038/s41598-019-38509-7, published online 12 February 2019

This Article contains a typographical error in the ‘Overall Amplifier Schematic’ section where,

“Full schematic and printed circuit board (PCB) fabrication data is available at http://www.fuw.edu.pl/mwinkowski/.”

should read:

“Full schematic and printed circuit board (PCB) fabrication data is available at http://www.fuw.edu.pl/~mwinkowski/.”

